# Identification of dynamic mass-action biochemical reaction networks using sparse Bayesian methods

**DOI:** 10.1371/journal.pcbi.1009830

**Published:** 2022-01-31

**Authors:** Richard Jiang, Prashant Singh, Fredrik Wrede, Andreas Hellander, Linda Petzold

**Affiliations:** 1 Department of Computer Science, University of California Santa Barbara, Santa Barbara, California, United States of America; 2 Department of Information Technology, Uppsala University, Uppsala, Sweden; University of Connecticut School of Medicine, UNITED STATES

## Abstract

Identifying the reactions that govern a dynamical biological system is a crucial but challenging task in systems biology. In this work, we present a data-driven method to infer the underlying biochemical reaction system governing a set of observed species concentrations over time. We formulate the problem as a regression over a large, but limited, mass-action constrained reaction space and utilize sparse Bayesian inference via the regularized horseshoe prior to produce robust, interpretable biochemical reaction networks, along with uncertainty estimates of parameters. The resulting systems of chemical reactions and posteriors inform the biologist of potentially several reaction systems that can be further investigated. We demonstrate the method on two examples of recovering the dynamics of an unknown reaction system, to illustrate the benefits of improved accuracy and information obtained.

## Author summary

Reconstructing the correlated reactions that govern a system of biochemical species from observational temporal data is an essential step in understanding many biological systems. To facilitate this process, we propose a robust, data-driven approach based on a sparse Bayesian statistical model. Our approach exploits sparse Bayesian priors and an unbiased observational model to recover a parsimonious, interpretable reaction system from mass-action relations, utilizing very little user input. On a set of simulated test problems, the method demonstrates increased robustness and decreased bias at different levels of measurement variability, while also producing interpretable reaction systems and quantifying uncertainty. As a tool, the approach can be used to flexibly interrogate biological systems while allowing incorporation of potentially uncertain domain knowledge to improve the efficiency and identifiability of the problem.

This is a *PLOS Computational Biology* Methods paper.

## Introduction

Developments in high-throughput experimental methodologies in biology have enabled the collection of massive amounts of time varying molecular data at small scales. This has resulted in significant advances in understanding the biochemical networks and mechanisms underlying physiological processes such as gene regulation. Indeed, greater understanding of regulatory processes at the single cell level can aid in the development of targeted therapies for diseases such as cancer [1–3]. A major challenge in this process is the translation of high-throughput, observational molecular data into analyzable and interpretable reaction networks. Typically, this is accomplished by utilizing significant biological insights to first define a reaction system, and then calibrating the model based on collected data, which while accurate, requires substantial time and effort to iterate. An appealing avenue is to utilize data-driven approaches for systems identification, whereby plausible biochemical reaction networks are generated and estimated directly from data without the need to initially propose a system. While recently, many such methods have been developed to infer networks from a wide variety of different datasets, it remains a challenging statistical and computational task [4]. Most works of estimating networks typically focus on either reconstructing a network without assuming any known dynamics due to destructive time series measurements [5–7], or producing networks that replicate dynamics, but without focusing on interpretability [8–12].

In this work, we are primarily interested on identifying interpretable mass-action biochemical reaction networks using only the observed time series of species concentrations. Expanding upon the problem formulation first proposed as Reactive SINDy in [13], we automatically enumerate the allowable mass-action reactions given a set of species and a library of ansatz reactions and utilize advances in sparse Bayesian inference to generate posterior distributions of interpretable biochemical reaction systems. Compared with Reactive SINDy, our method provides uncertainty estimates over potential reaction systems, reduces a major source of bias in the previous method, and produces potentially several interpretable reaction networks. Furthermore, the transparent statistical formulation of the problem allows us to easily incorporate existing, potentially uncertain, domain knowledge via prior distributions to improve the efficiency and identifiability of the problem.

The remainder of this paper proceeds as follows. In Materials and methods, we describe mass-action biochemical reaction networks and formulate the problem of inferring these networks from observational data. Next, we propose improvements to the existing methodology and describe the specifics of the proposed model applied to inference of reaction networks. In Results, we demonstrate how our methodology can be used in two different examples to retrieve interpretable networks from observational concentration data. We close in Discussion by noting a few details for usage, detailing some future directions, and mentioning the limitations of our method. All implementations and code can be found at https://github.com/rmjiang7/bayes_reactive_sindy.

## Materials and methods

### Mass-action biochemical kinetics

Systems of biochemical species reacting under any number of reaction channels are commonly modeled dynamically using the framework of chemical kinetics. Specifically, denote $X\left(t\right)\in R^{N}$ as the vector of concentrations of each of *N* species at time *t*. The evolution of the system can be modeled using the following set of coupled ordinary differential equations (ODEs) formally known as the reaction rate equations:

$$\begin{array}{c} \frac{dX}{dt}=Sf\left(X\right), \end{array}$$ (1)

where $S\in Z^{N\times M}$ is the stoichiometric matrix with *M* reactions among *N* species and **f(X)** is the vector of all rate functions.

Although theoretically, **f(X)** can take the form of any nonlinear function, in this work we assume that the system follows mass-action kinetics [14] and thus, the reaction rates are proportional to the product of the concentrations of each reactant in the case of multiple reactants, and proportional to the concentration of the reactant in single reactant reactions.

### Network inference for mass-action reaction systems

Suppose we observe a time series of *N* species concentrations at *T* discrete times:

$$\begin{array}{c} \hat{X}\left(t_{j}\right)\in R^{N},\;j=\left\{0,\ldots ,T\right\}. \end{array}$$

Given this data and assuming that the system is governed by up to 2nd-order mass-action kinetics and the dynamics of Eq (1), we wish to recover a parsimonious system of expressible reactions that can explain the observed data.

Under these constraints, this problem can be posed as a linear regression, given a library of ansatz reactions. More specifically, suppose we initially specify a large set of *D* possible reactions among the *N* species in our system. Each reaction can be expanded into a stoichiometry *s* and a rate function *f*(**X**), where the rate function is known due to the assumption of mass-action rate kinetics. Let *S*_*c*_ ∈ *Z*^*N*×*D*^ denote the complete stoichiometric matrix constructed by stacking all *D* stoichiometries into a matrix. The reaction rate equations then take the form,

$$\begin{array}{c} \frac{dX}{dt}=S_{c}^{T}(\begin{array}{c} k_{1}f_{1}\left(X\right)\\ k_{2}f_{2}\left(X\right)\\ \cdots \\ k_{D}f_{D}\left(X\right) \end{array}), \end{array}$$ (2)

where *k*_*i*_ > 0 is the unknown rate-constant and *f*_*i*_ is simply a product of the reactants for the *i*-th reaction. Letting **k** = [*k*_1_, …, *k*_*D*_] denote the vector of all of the reaction rates, we aim to estimate **k** such that, when solved, Eq (2) replicates the observations $\hat{X}$ at all *t*_*j*_. Many methods exist to solve these types of problems, such as ridge, LASSO, and Elastic net regression [15–18].

Although *S*_*c*_ is potentially high dimensional, conditional on the initially specified set of *D* reactions, in most situations *D* over-specifies the possible reactions. Hence, to replicate the observations, a safe assumption is that most potential reactions do not exist, which is equivalent to setting *k*_*i*_ = 0 when the *i*-th reaction does not contribute to the dynamics of the system. This assumption can be captured by estimating **k** using sparse regression methods. A small reaction system can then be expressed by rewriting the system in terms of only the non-zero reactions.

Sparse regression methods for estimating dynamical systems from data have been widely applied in the last few years. More generally, when Eq (1) is generated from polynomial basis functions rather than ansatz reactions, this becomes Sparse Identification of Nonlinear Dynamics (SINDy) [19], which has been applied to biological systems [8], though without the specific aim to recover interpretable reactions. Reactive SINDy, as described above, expands SINDy by constraining the basis functions to such ansatz mass-action reactions. Both of these methods estimate the coefficients **k** using LASSO regularization, resulting in maximum likelihood networks that do not inform about the uncertainty associated with the particular fits, an especially important feature when data is sparse and noisy. Reactive SINDy uses finite difference derivative estimates from observations to transform Eq (2) into a linear regression problem, which can result in significant bias for estimating networks when measurements are sparse and noisy, as is often the case in biological systems.

More specifically, using the assumptions of mass-action kinetics and the law of parsimony, Reactive SINDy solves a mixed LASSO and ridge regression optimization problem. Letting $\frac{d\hat{X}}{dt}\left(t_{i}\right)$ be the derivatives numerically estimated from the observations $\hat{X}\left(t_{j}\right)$ via second-order finite differences, the optimization problem solved is

$$\begin{array}{cc} \Phi (X) & =(\begin{array}{c} k_{1}f_{1}\left(X\left(t_{j}\right)\right)\\ k_{2}f_{2}\left(X\left(t_{j}\right)\right)\\ \cdots \\ k_{D}f_{D}\left(X\left(t_{j}\right)\right) \end{array})\\ k=\text{arg}\;\underset{k}{\text{min}} & \left(\frac{1}{2T}{\left\|\hat{X}-\Phi \left(X\right)\right\|}_{F}^{2}+\alpha \lambda {\left\|k\right\|}_{2}+\alpha \left(1-\lambda \right){\left\|k\right\|}_{2}^{2}\right)\\ & \;\text{subject}\;\text{to}\;k\geq 0. \end{array}$$

The equivalent statistical model for the LASSO optimization can be summarized as

$$\begin{array}{cc} k_{i} & ∼\text{Laplace}(\lambda ),\\ \frac{d\hat{X}}{dt}\left(t_{j}\right)∼\text{Normal} & (S_{c}^{T}(\begin{array}{c} k_{1}f_{1}\left(X\left(t_{j}\right)\right)\\ k_{2}f_{2}\left(X\left(t_{j}\right)\right)\\ \cdots \\ k_{D}f_{D}\left(X\left(t_{j}\right)\right) \end{array}),1),\;j=0,\ldots ,T, \end{array}$$ (3)

which can also be fit using Bayesian methods to provide uncertainty estimates.

In this work we improve on Reactive SINDy in two key ways. First, we estimate **k** using the sparse Bayesian regularized horseshoe prior to obtain uncertainty estimates as well as to introduce a natural way of incorporate existing domain knowledge via prior distributions. Second, we avoid biased numerical derivative estimates by re-formulating the statistical model in terms of the solution of the ODE. This better captures the observational model and allows us to incorporate alternative models of measurement noise. Using recent advances in automatic differentiation software for sensitivity analysis of ODE systems [20–23], this can be solved efficiently and provides more accurate solutions, especially in the case of sparsely measured data.

### Bayesian reactive SINDy

In this section we introduce the regularized horseshoe prior [24] used in our Bayesian formulation of the Reactive SINDy model and the modified observational model, which better captures the measurement process and avoids biased, low-order derivative estimates. We construct the complete stoichiometric matrix *S*_*c*_ using a library of possible mass-action ansatz reactions and all reaction rates are specified by **k** as indicated in Eq (2). Details for how we construct a set of ansatz reactions can be found in the S1 Appendix.

#### Sparse Bayesian regularized horseshoe priors

A challenge in implementing a Bayesian formulation of this problem is the fact that the LASSO penalization used for sparse parameter estimation, which can be translated as a statistical model to Eq (3), does not result in sparse Bayesian posterior distributions. Instead, we adapt the regularized horseshoe prior, an extension of the standard horseshoe prior [25], which is a drop-in replacement for the LASSO derived Laplace prior.

Letting *N* be the number of species, *T* be the number of observations, and *D* be the number of ansatz reactions, the regularized horseshoe prior placed on the reaction coefficients **k** takes the form

$$\begin{array}{cc} \lambda_{i} & ∼{\text{Cauchy}}^{+}\left(0,1\right),\\ \tilde{\lambda_{i}} & =\sqrt{\frac{c^{2}\lambda_{i}^{2}}{c^{2}+{\left(\tau \lambda_{i}\right)}^{2}}},\\ k_{i} & ∼\text{Normal}(0,\tau \tilde{\lambda_{i}}),\;i=1,\ldots ,D. \end{array}$$ (4)

This promotes sparse solutions in the following way: each reaction rate *k*_*i*_ is given a normal prior centered around 0 with a standard deviation of *τ*λ_*i*_, where *τ* is a global shrinkage parameter shared among all reaction rates and λ_*i*_ is a positive parameter specific to each reaction rate. The heavy tailed half-Cauchy priors on the individual λ_*i*_ allows for the values to grow extremely large. This has the following effect:

$$\begin{array}{cc} \text{if}\;{\left(\tau \lambda_{i}\right)}^{2}\gg c^{2}, & \;\tau \tilde{\lambda_{i}}\to c\\ \text{if}\;{\left(\tau \lambda_{i}\right)}^{2}\ll c^{2}, & \;\tau \tilde{\lambda_{i}}\to \tau \lambda_{i}. \end{array}$$

Thus, if *k*_*i*_ is estimated to be non-zero, λ_*i*_ is allowed to become large and *k*_*i*_ breaks away from *τ* toward a regularized value of *c*^2^, which is an estimate of the scale of the non-zero terms. On the other hand, if *k*_*i*_ is estimated to be zero, λ_*i*_ becomes small and *k*_*i*_ is shrunken to 0 with an often very small standard deviation. The horseshoe prior has the effect of placing significant prior mass towards 0 for all parameters, but allowing for any individual parameter to be non-zero if there is sufficient evidence to do so. The regularized horseshoe further shrinks non-zero estimates using a Gaussian slab with variance *c*^2^, to help when parameters are weakly identified and to prevent non-zero values from growing too large.

The pivotal global shrinkage parameter *τ* specifies the scale of the near-zero reaction rates, which is relevant because, compared to the spike-and-slab prior [26], the regularized horseshoe prior is continuous in all parameters, preventing any parameter from becoming exactly 0. Furthermore, smaller values of *τ* also result in sparser networks. For our problem, as reaction rates can often be very small, specifying the scale at which a reaction is considered negligible can dramatically affect the interpretation and the simulated dynamics.

Following [24], we place a hyper-prior on the term *c* with distribution

$$\begin{array}{c} c∼\text{Inv-Gamma}(a,b). \end{array}$$

The *c* parameter regularizes by essentially placing a $N(0,c^{2})$ prior on non-zero rates, preventing them from getting too large. The non-regularized horseshoe is retrieved when *c*^2^ → ∞.

The regularized horseshoe prior offers a few distinct advantages compared to other sparse Bayesian priors. Primarily, the dependency structure formed by introducing the global *τ* and the local λ_*i*_ parameters leads to sparser solutions that can borrow information from other reactions. The regularized horseshoe, as a continuous relaxation of the commonly used sparse Bayesian spike-and-slab prior [26, 27], allows for efficient Bayesian computation using modern gradient based MCMC samplers such as Hamiltonian Monte Carlo (HMC) [28] or Variational Inference [29]. This allows it to be implemented in probabilistic programming languages such as Stan [30], PyMC3 [31], or Pyro [32].

#### Observational model

A potentially large source of bias in SINDy and Reactive SINDy as presented in Eq (3) is the need to first estimate $\frac{d\hat{X}}{dt}$ from observations of the system. This presents an issue as standard methods of estimating derivatives, such as finite difference methods, become much less accurate as the time between observations increases, resulting in heavily biased estimates of **k**. To correct for this, we modify the observational model as follows:

$$\begin{array}{cc} Z(t_{j}) & =\int_{0}^{t_{j}}S_{c}^{T}(\begin{array}{c} k_{1}f_{1}\left(Z\left(t\right)\right)\\ k_{2}f_{2}\left(Z\left(t\right)\right)\\ \cdots \\ k_{D}f_{D}\left(Z\left(t\right)\right) \end{array})dt\\ \hat{X}\left(t_{j}\right) & ∼\text{Log-Normal}(Z\left(t_{j}\right),\sigma ),\;j=0,\ldots ,T. \end{array}$$

Rather than assuming that we observe derivatives of the process, as in the Eq (3), this formulation models that the underlying system follows a latent variable *Z*(*t*_*j*_), which is the solution of the ODE. We observe noisy measurements of the underlying system $\hat{X}\left(t_{j}\right)$ at times *t*_*j*_. By directly modeling the observations, there is no need to pre-process the data by estimating derivatives.

In this work, we also assume that the measured concentrations of each species are corrupted by log-normal error. This captures both that concentration measurements are strictly positive and that at higher concentrations, measurements are more variable. In addition, this formulation enables us to easily change the measurement error model to better capture the user’s beliefs, without modifying the regularized horseshoe prior for inferring the network. As an example, a Poisson error model, such as that explained in [33], can be applied under the assumption that measured values are positive and discrete, and that measurements at some time *t*_*j*_ are distributed with mean and variance of *Z*(*t*_*j*_). The use of MCMC for sampling enables the observational model to be configured based on the experimental setup as long as the likelihood remains tractable.

The use of MCMC for sampling enables the observational model to be configured based on the experimental setup as long as the likelihood remains tractable. For biochemical reaction networks, PTLasso [34] apply a similar latent observation model, but with the Laplace prior to the parameters of a biochemical reaction network, further using parallel tempering MCMC to obtain sparse Bayesian estimates on models of up to a dozen different reactions.

The latent variable formulation also allows for the realistic scenario of observing only some of the species in the system. Suppose that in a system consisting of 5 species, we can only observe species *n* = {1, 2}. Then the observational model can be easily modified to

$$\begin{array}{cc} Z(t_{j}) & =\int_{0}^{t_{j}}S_{c}^{T}(\begin{array}{c} k_{1}f_{1}\left(Z\left(t\right)\right)\\ k_{2}f_{2}\left(Z\left(t\right)\right)\\ \cdots \\ k_{D}f_{D}\left(Z\left(t\right)\right) \end{array})dt\\ \hat{X_{n}}\left(t_{j}\right)∼ & \text{Log-Normal}\left(Z_{n}\left(t_{j}\right),\sigma \right),\;j=0,\ldots ,T,\;n=\left\{1,2\right\}. \end{array}$$ (5)

This is possible because the latent trajectory *Z* does not directly depend on the observed values $\hat{X}$. In comparison, for Eq (3) used in Reactive SINDy and SINDy, the regression directly depends on the observed values, which thus requires complete observations of the system.

#### Statistical model and estimation

Combining the regularized horseshoe prior and the latent variable observational model, the complete hierarchical statistical model is specified by

$$\begin{array}{cc} \lambda_{i} & ∼{\text{Cauchy}}^{+}\left(0,1\right),\\ \tilde{\lambda_{i}} & =\sqrt{\frac{c^{2}\lambda_{i}^{2}}{c^{2}+{\left(\tau \lambda_{i}\right)}^{2}}},\\ k_{i} & ∼\text{Normal}(0,\tau \tilde{\lambda_{i}}),\;i=1,\ldots ,D\\ Z(t_{j}) & =\int_{0}^{t_{j}}S_{c}^{T}(\begin{array}{c} k_{1}f_{1}\left(Z\left(t\right)\right)\\ k_{2}f_{2}\left(Z\left(t\right)\right)\\ \cdots \\ k_{D}f_{D}\left(Z\left(t\right)\right) \end{array})dt\\ \hat{X}\left(t_{j}\right) & ∼\text{Log-Normal}(Z\left(t_{j}\right),\sigma ). \end{array}$$ (6)

The algorithm for network identification is then as follows. First, we construct the complete stoichiometric matrix, *S*_*c*_, and the set of linear and nonlinear reaction rate functions **f(X)** implied by mass-action kinetics. In our examples, the library of reactions consists of a large set of zero, first, and second order reactions types between all species modeled, which is automatically defined by our implementation. We note here that our method of generating possible reactions is intended to be general to demonstrate the method. In practice, the set of possible reactions is something the modeler can and should modify according to the constraints of the problem.

Provided with *S*_*c*_ and **f(X)**, the sparse Bayesian posterior distribution $p(k|\hat{X})$ is approximated from the above statistical model using the No-U-Turns [35] sampler implemented in Stan [30].

As the regularized horseshoe is continuous in all parameters, no rate parameter will be set exactly to zero. Thus to decide whether a reaction is to be removed from the system, we employ the pruning technique adopted from [36]. Specifically, we estimate

$$\begin{array}{c} P\left(\tau \tilde{\lambda_{i}}<\delta \right)>p_{0},\;i=1,\ldots ,D \end{array}$$

using the posterior distribution. This can be roughly interpreted as pruning all reactions where the posterior probability that the scale of *k*_*i*_ is less than *δ* is sufficiently large. This metric is sensible because rates which are shrunken towards 0 in the regularized horseshoe are scaled by *τ*λ_*i*_. This leaves two tuning hyperparameters, *δ* and *p*_0_. These can be calibrated for a model by choosing the threshold such that, allowing more reactions does not improve the model’s fit to the data, while removing reactions degrades the fit. In our examples, we find that *δ* = 1*e*^−3^ and *p*_0_ = 0.90 work well for these models.

The complete implementation and all replicating results can be found at https://github.com/rmjiang7/bayes_reactive_sindy.

## Results

We demonstrate our method on two synthetic examples where data is first generated from a known system of reactions and our method is used to recover the underlying network from a relatively large set of possible reactions. In each example, results are compared to those of Reactive SINDy, to show the ability of our model to obtain a network, with uncertainty estimates, that replicates the observations in addition to demonstrating the superior performance in the case of sparse observations due to the modified observational model. In the second, larger problem, we demonstrate the ability of our method to discover multiple small reaction systems that can capture the observations and discuss identifiability issues. Further descriptions and more precise model specifications can be found in the S1 Appendix.

### Lotka-Volterra

The Lotka-Volterra predator-prey system is a simple but informative example of a non-linear system with oscillatory dynamics. Although not strictly a biochemical reaction system, we provide it as an example for evaluating the model formulation and method. Briefly, the Lotka-Volterra system models the interaction dynamics of two species *X* ≔ {*P*, *Y*} where *P* is the predator and *Y* is the prey. This can be described using the following reactions:

$$\begin{array}{cc} Y & \overset{k_{1}}{\to }2Y\\ P+Y & \overset{k_{2}}{\to }2P\\ P & \overset{k_{3}}{\to }\phi , \end{array}$$

which corresponds to the following stoichiometric matrix and rate vectors under mass-action kinetics,

$$\begin{array}{c} S=(\begin{array}{cc} 0 & 1\\ 1 & 0\\ 0 & -1 \end{array}),\;f\left(X\right)=(\begin{array}{c} k_{1}\left[Y\right]\\ k_{2}\left[P\right]\left[Y\right]\\ k_{3}\left[P\right] \end{array}). \end{array}$$

With *k*_1_ = 1, *k*_2_ = 0.01, *k*_3_ = 0.3 and initial conditions *X*(*t*_0_) ≔ {50, 100}, this gives rise to stable oscillations.

**Fig 1:**
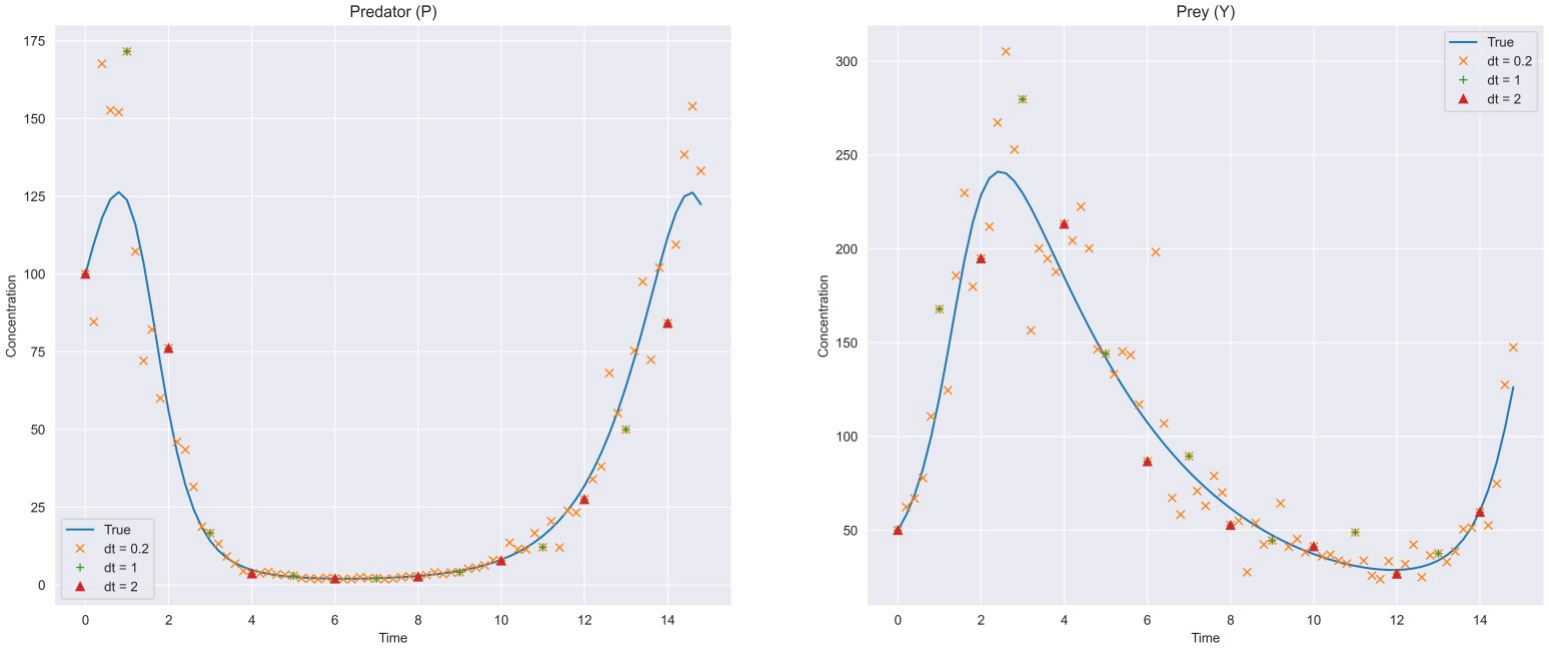
Lotka-Volterra observation data. Simulated data used for network identification. Log-Normal noise is added to the true trajectory, and measurement frequency is changed to show the uncertainty in posteriors.

Data is generated by solving the above system of reactions and perturbing with Log-Normal(0, 0.2) noise at fixed times to simulate a noisy measurement process. The reactions comprising the complete stoichiometric matrix *S*_*c*_ from which we will recover the underlying system is provided in Table 1 and adopted from [13]. In total, there are 16 possible reactions in this system, three of which are non-zero in the original system. We generate data at three different measurement frequencies dt = {0.2, 1, 2} between *t* = [0, 15] and estimate **k** separately for each using the same *S*_*c*_. Trajectories of the two species are shown in Fig 1. For estimation from the regularized horseshoe model, we set *τ* = 1*e*^−8^ and estimate *c* along with the other parameters by placing the prior *c* ∼ Inv- Gamma(4, 4). A total of 4000 samples are drawn using four MCMC chains. We note that while we use MCMC for accuracy and demonstration purposes, variational inference can also be used to obtain fast approximate solutions and is supported in our implementations. In our experiments, we found that the variational approximations were generally reliable, though this largely depends on the problem.

**Table 1:** Library of ansatz reactions for the Lotka-Voltera model.

Reaction Index	Allowed Reactions	True Rate Constant
0	$2X\overset{k_{1}}{\to }0$	*k*_1_ = 0
1	$2Y\overset{k_{2}}{\to }0$	*k*_2_ = 0
2	$X\overset{k_{3}}{\to }2X$	**k_3_ = 1.0**
3	$X+Y\overset{k_{4}}{\to }2Y$	**k_4_ = 0.01**
4	$X\overset{k_{5}}{\to }0$	**k_5_ = 0.3**
5	$X+Y\overset{k_{6}}{\to }2X$	*k*_6_ = 0
6	$X\overset{k_{7}}{\to }0$	*k*_7_ = 0
7	$2Y\overset{k_{8}}{\to }Y$	*k*_8_ = 0
8	$Y\overset{k_{9}}{\to }2Y$	*k*_9_ = 0
9	$2X\overset{k_{10}}{\to }X$	*k*_10_ = 0
10	$X+Y\overset{k_{11}}{\to }X$	*k*_11_ = 0
11	$X+Y\overset{k_{12}}{\to }Y$	*k*_12_ = 0
12	$2X\overset{k_{13}}{\to }Y$	*k*_13_ = 0
13	$X\overset{k_{14}}{\to }Y$	*k*_14_ = 0
14	$Y\overset{k_{15}}{\to }X$	*k*_15_ = 0
15	$X\overset{k_{16}}{\to }2Y$	*k*_16_ = 0

**Fig 2:**
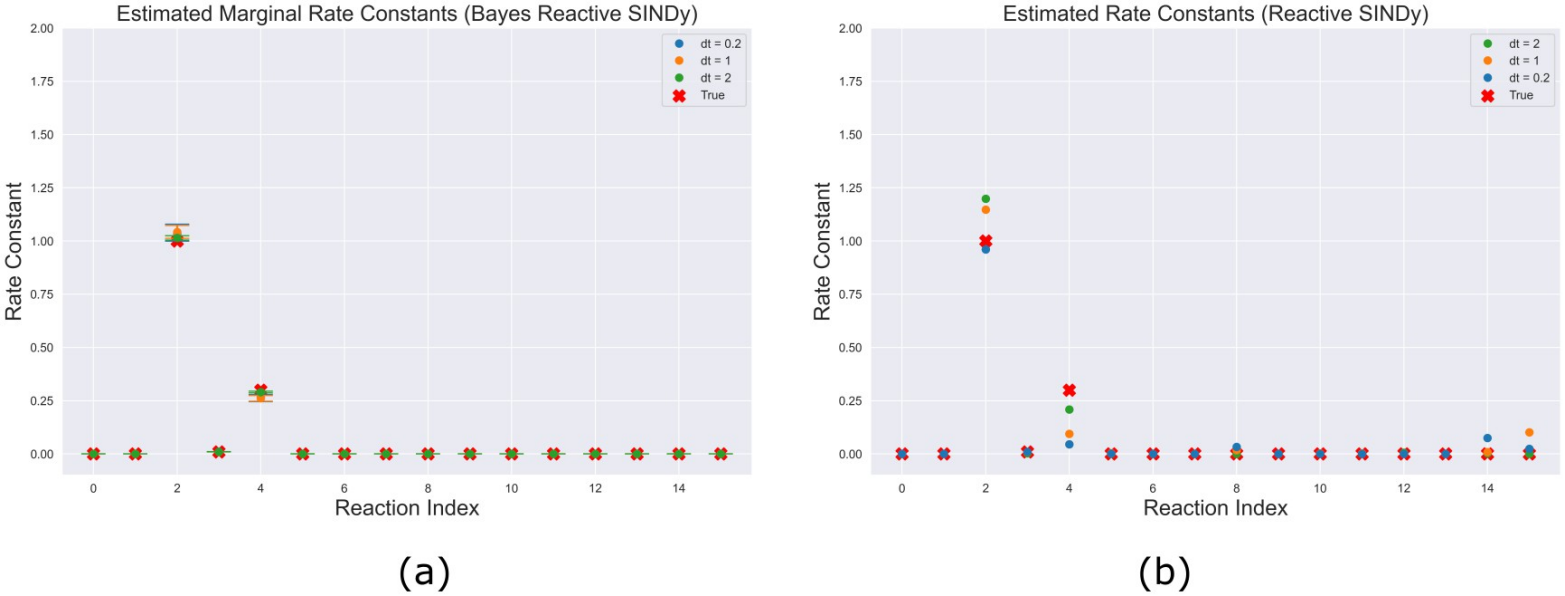
Lotka-Volterra estimated reactions. (a) Estimated parameters using Eq (6). Reactions correspond to the reactions specified in Table 1 (b) Estimated parameters using Reactive SINDy at different measurement frequencies as well as using noise-less measurements.

In Fig 2a, we show the posterior credible intervals for the recovered rate constants from each of the three measurement frequencies, which are heavily centered around the true values for all reactions. Fig 2b shows the point estimates obtained by using Reactive SINDy under equivalent experimental setups. Notably, both methods can recover the reaction system with frequent measurements but Reactive SINDy degrades considerably as measurements become more sparse.

**Fig 3:**
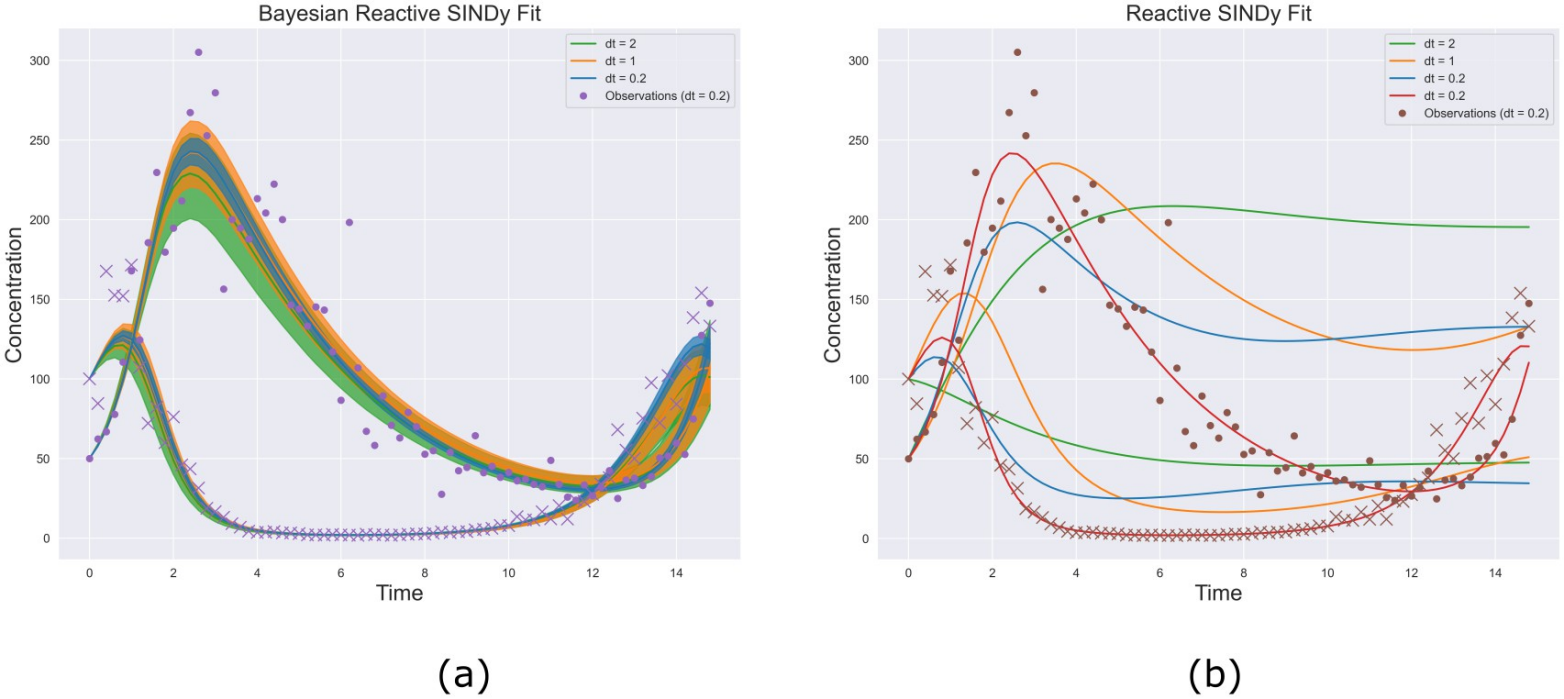
Reconstructed trajectories. (a) Using posterior samples from Eq (6). Even at smaller observation frequencies, the observed data is accurately captured, though (as expected) with greater uncertainty. (b) As Reactive SINDy estimates derivatives, errors in the numerical methods lead to large deviations in the reconstructed trajectories as sampling frequency and noise increase. Although a single trajectory at dt = 0.2 may capture the oscillating behavior, it is clearly biased away from the true observations.

More specifically, the difference in the results demonstrates the bias introduced by estimating derivatives. At observation intervals dt = 1.0 and dt = 2.0, too much information is lost from estimating derivatives coupled with measurement noise to obtain the correct system. Fig 3 shows the differences in inferred dynamics along with predictive uncertainty intervals from the networks recovered using our observational model, (a), and Reactive SINDy, (b). Our model remains in phase with the observations while the networks derived from using estimated derivatives demonstrate a systematic bias away from the true reaction system, even in the case of dt = 0.2 due to measurement noise.

**Fig 4:**
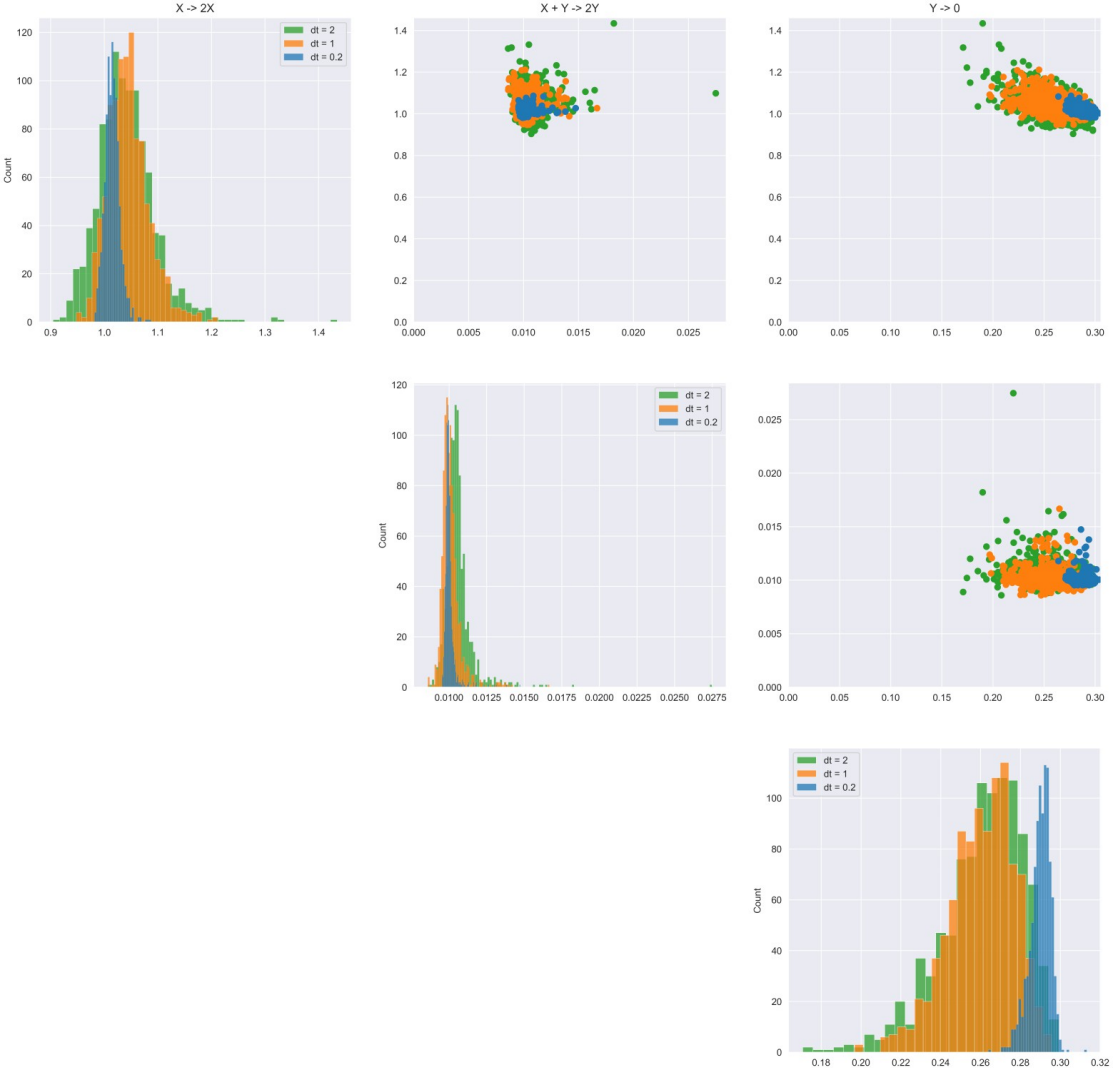
Lotka-Volterra: Posterior distributions of non-zero reactions using the proposed model. As expected, uncertainty in the parameters decreases as the measurement frequency decreases, but all are concentrated in relatively the same area. Only a single network is consistently identified given this data, indicating that identifiabiltiy is not a problem for this system.

With the Bayesian treatment of the problem, we can also quantify uncertainty in the non-zero reaction rates. This informs us of the plausible range of reaction rates, given the observed data, and can be useful to detect which parameters the model is able to identify with evidence of correlated reactions. In Fig 4, the posterior distributions of the non-zero estimated parameters are shown, demonstrating that as we increase measurement frequency, uncertainty decreases. Furthermore, in this system there is mild correlation between the reaction rates, indicating that they vary together to replicate the oscillating behavior.

#### Partially observed species

In the previous example, we assumed that the species were completely observed. However, under the latent variable formulation, this is not strictly required. In this section, we demonstrate inference of the network for the identifiable Lotka-Volterra example, when only the prey species, *Y*, is observed. In this case, the statistical observational model can be changed to,

$$\begin{array}{cc} Z(t_{j}) & =\int_{0}^{t_{j}}S_{c}^{T}(\begin{array}{c} k_{1}f_{1}\left(Z\left(t\right)\right)\\ k_{2}f_{2}\left(Z\left(t\right)\right)\\ \cdots \\ k_{D}f_{D}\left(Z\left(t\right)\right) \end{array})dt\\ \hat{Y}\left(t_{j}\right)∼ & \text{Log-Normal}(Z_{2}\left(t_{j}\right),\sigma ),\;j=0,\ldots ,T, \end{array}$$ (7)

where we apply the likelihood only to the observations of *Y*.

**Fig 5:**
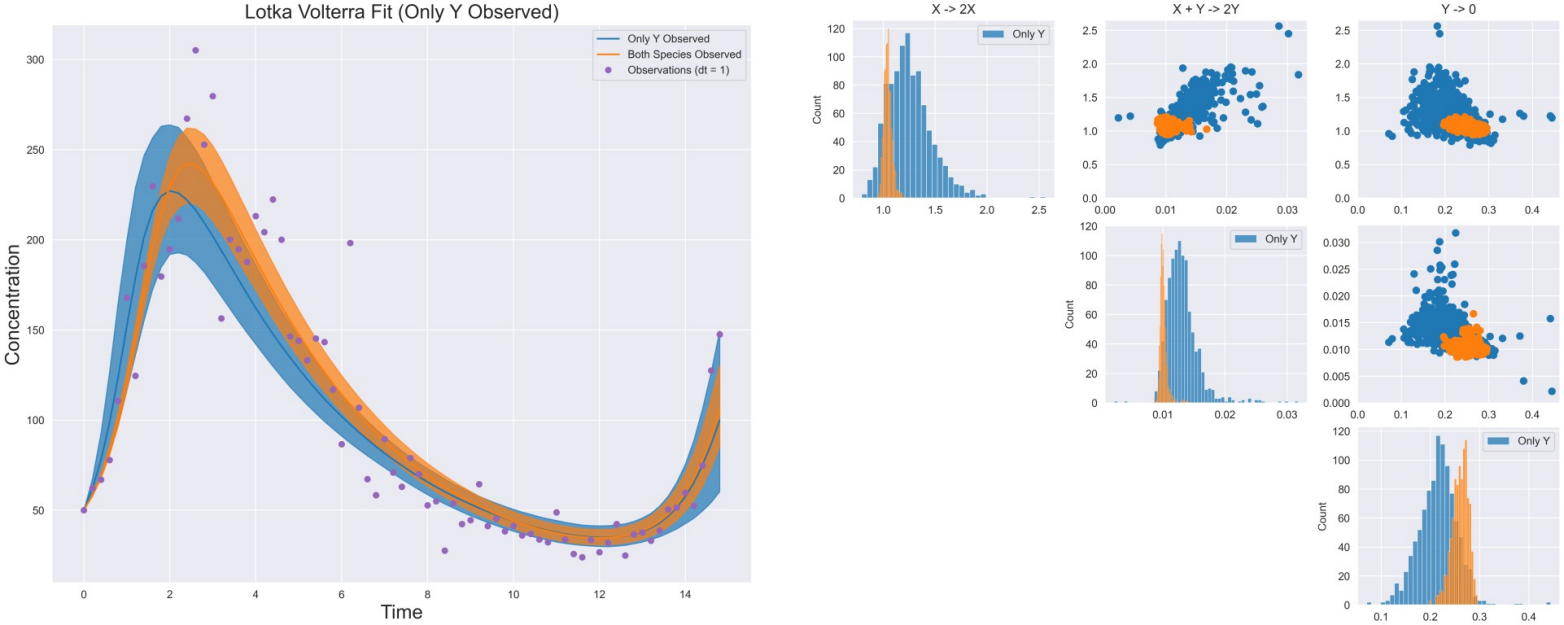
Identified trajectories and posterior from partial observations. The true network can still be captured using only observations of *Y* however the credible intervals are significantly higher due to the loss of observations of *P*.

Fig 5 shows the simulated trajectories and posterior distributions obtained by using our model under this scenario. Compared to the situation where both species are observed, the uncertainty is significantly higher for the same reactions because the information gained from observing *P* is lost. However, the method is still able to retrieve the correct networks, as the oscillating regime for this problem is generally unique.

#### Sums of observed species

Similar to above, the latent variable formulation we have presented allows for modeling of the situation where a sum of species concentrations is observed, but not any of the individual species. In this case, for the Lokta-Volterra system, letting *W* = *X* + *Y* be the observed sum of *X* and *Y*, the statistical model can be stated as

$$\begin{array}{cc} Z(t_{j}) & =\int_{0}^{t_{j}}S_{c}^{T}(\begin{array}{c} k_{1}f_{1}\left(Z\left(t\right)\right)\\ k_{2}f_{2}\left(Z\left(t\right)\right)\\ \cdots \\ k_{D}f_{D}\left(Z\left(t\right)\right) \end{array})dt\\ \hat{W}\left(t_{j}\right)∼ & \text{Log-Normal}(Z_{1}\left(t_{j}\right)+Z_{2}\left(t_{j}\right),\sigma ),\;j=0,\ldots ,T, \end{array}$$ (8)

**Fig 6:**
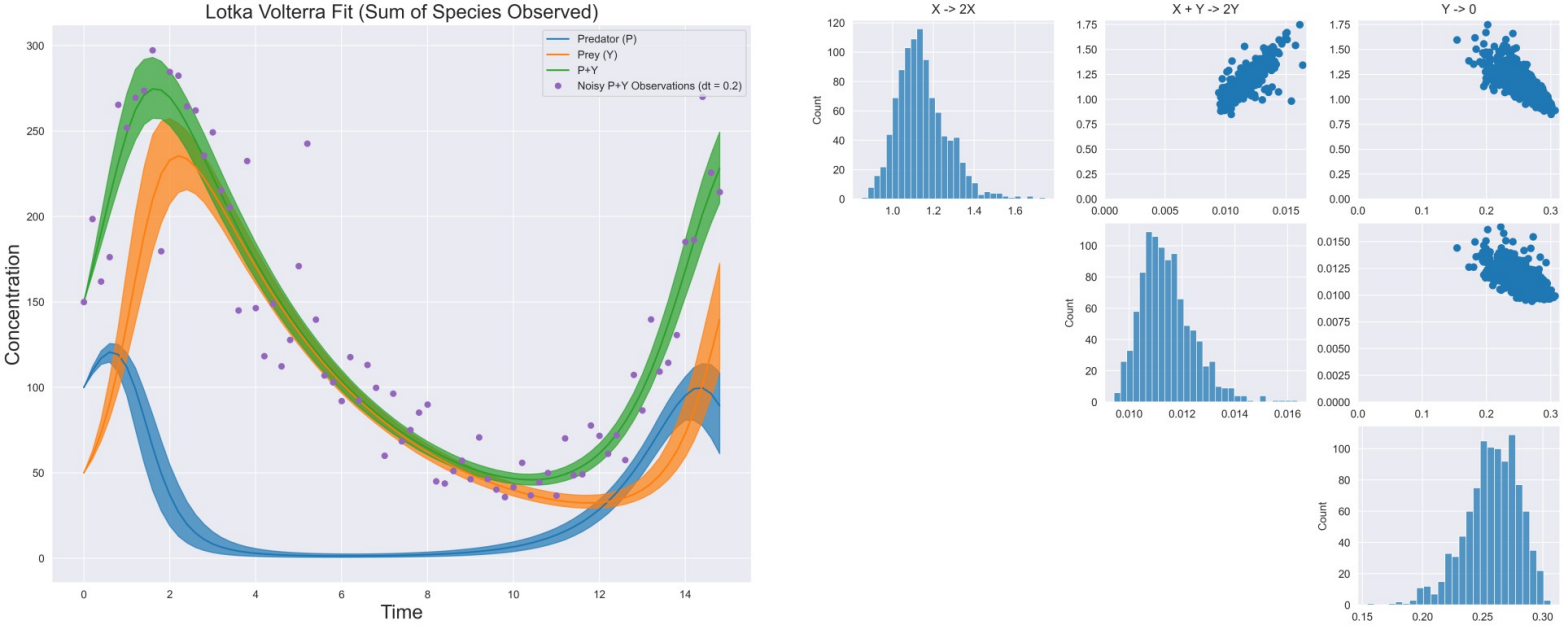
Identified trajectories and posterior from additive. Similar to the case of observing only one *Y*, the true network can still be recovered in this example however credible intervals are significantly larger.

Fig 6 shows that our model under only additive observations can still recover the correct network under this highly identifiable model. Similar to the previous case, uncertainties in the rate constants are, as expected, larger.

### Prokaryotic auto-regulation

**Fig 7:**
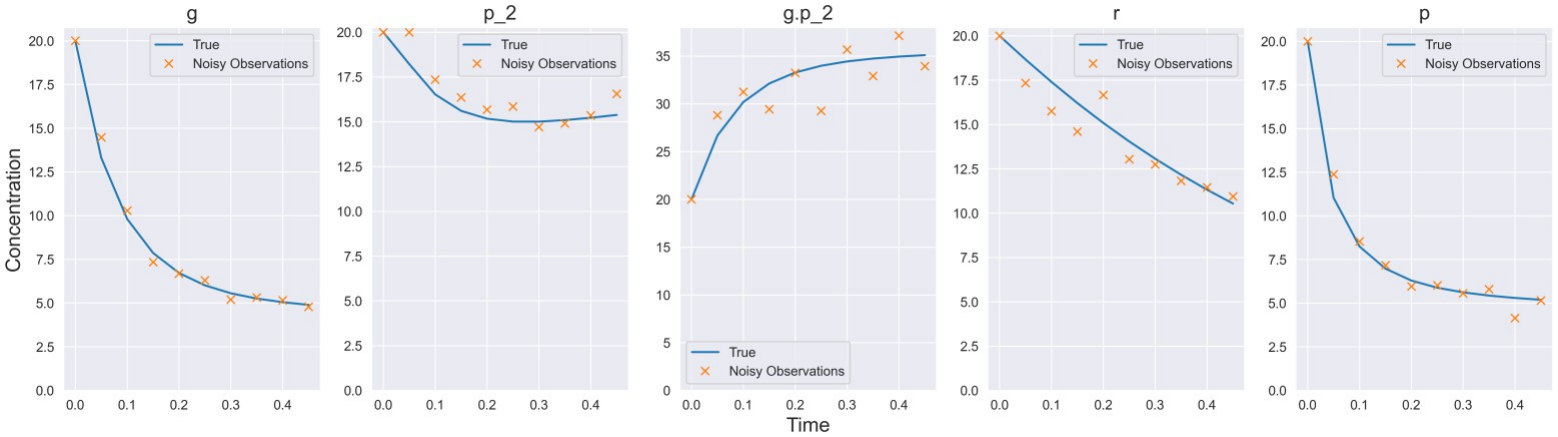
Prokaryotic auto-regulation observation data. Simulated data used for the prokaryotic auto-regulation model. Log-normal observational noise is added to the true trajectory.

To evaluate the method on a larger reaction system with more possible reactions, we explore a simple synthetic model of auto-regulation of a protein *P* by a gene *g* in a prokaryotic cell [37]. The model is described by the following reaction system:

$$\begin{array}{cc} g+P_{2} & \overset{k_{1}}{\to }gP_{2}\;(\text{Repression})\\ gP_{2} & \overset{k_{2}}{\to }g+P_{2}\\ g & \overset{k_{3}}{\to }g+r\;(\text{Transcription})\\ r & \overset{k_{4}}{\to }r+P\;(\text{Translation})\\ 2P & \overset{k_{5}}{\to }P_{2}\;(\text{Dimerization})\\ P_{2} & \overset{k_{6}}{\to }2P\\ r & \overset{k_{7}}{\to }\phi \;(\text{mRNA}\;\text{Degradation})\\ P & \overset{k_{8}}{\to }\phi \;(\text{Protein}\;\text{Degradation}), \end{array}$$

where *gP*_2_ is the bound gene and *r* is the mRNA of protein *P*. Protein *P* represses its own transcription by binding to an available gene location. Denoting *X* ≔ {*g*, *P*_2_, *gP*_2_, *r*, *P*}, we generate data from the system with parameters *k*_1_ = 0.5, *k*_2_ = 1, *k*_3_ = 0.15, *k*_4_ = 1, *k*_5_ = 0.5, *k*_6_ = 0.5, *k*_7_ = 1.5, *k*_8_ = 0.3 and initial conditions *X*(*t*_0_) ≔ {20, 20, 20, 20, 20, 20} at dt = 0.05 for times in the interval [0, 0.5]. Furthermore, Log-Normal(0, 0.07) noise is added to the observations. At these parameter values, *g* and *P* decay rapidly, thus a small *dt* is required to provide sufficient information to the model. True trajectories and observations are shown in Fig 7.

Using our library of ansatz reactions, we construct a complete stoichiometric matrix *S*_*c*_ of 260 possible reactions. The exact reactions included can be explored in the code repository. For estimation from the regularized horseshoe model in this problem, we set *τ* = 1*e*^−6^ and estimate *c* along with the other parameters by setting *c* ∼ Inv- Gamma(5, 25). We run several MCMC chains to obtain results however only report the best two networks obtained for each experiment.

#### Including known reactions

To replicate the more common situation where the biologist has prior domain knowledge about the system under study, we explored the scenario where the first 4 reactions and rate parameters, *k*_1_, *k*_2_, *k*_3_, and *k*_4_, are known with confidence and the aim is to retrieve a system of reactions which replicates the observations, given these four known reactions. Below, we present the results of this setting to demonstrate a realistic situation where partial knowledge about system. The same experiment when no reactions are known is presented in the S1 Appendix with similar results though converging to different sparse networks.

Table 2 lists the two selected networks obtained from MCMC chains with the rate constants set to the posterior median. Notably, each chain converges to different reaction pathways, neither of which are the true generating network. We note that, though we only present two networks here, our method was capable of producing several different reaction pathways with roughly the same number of reactions also capable of capturing the data.

**Table 2:** Selected recovered networks for prokaryotic auto-regulation system. The first 4 reactions are assumed to be known and the remaining reactions are to be inferred by the method.

True Network	Network 1	Network 2
$g+P_{2}\overset{0.5}{\to }{\text{gP}}_{2}$	$g+P_{2}\overset{0.5}{\to }{\text{gP}}_{2}$	$g+P_{2}\overset{0.5}{\to }{\text{gP}}_{2}$
${\text{gP}}_{2}\overset{1}{\to }g+P_{2}$	${\text{gP}}_{2}\overset{1}{\to }g+P_{2}$	${\text{gP}}_{2}\overset{1}{\to }g+P_{2}$
$g\overset{0.15}{\to }g+r$	$g\overset{0.15}{\to }g+r$	$g\overset{0.15}{\to }g+r$
$r\overset{1}{\to }r+P$	$r\overset{1}{\to }r+P$	$r\overset{1}{\to }r+P$
$2P\overset{0.5}{\to }P_{2}$	$2r\overset{0.05}{\to }P$	$2P\overset{0.5}{\to }P_{2}$
$P_{2}\overset{0.5}{\to }2P$	$2P\overset{0.26}{\to }gP_{2}$	$2P_{2}\overset{0.06}{\to }P$
$r\overset{1.5}{\to }\phi$	$P_{2}+gP_{2}\overset{0.04}{\to }P$	$gP_{2}+r\overset{0.05}{\to }P_{2}+gP_{2}$
$P\overset{0.3}{\to }\phi$	$P_{2}+P\overset{0.4}{\to }2P_{2}$	

**Fig 8:**
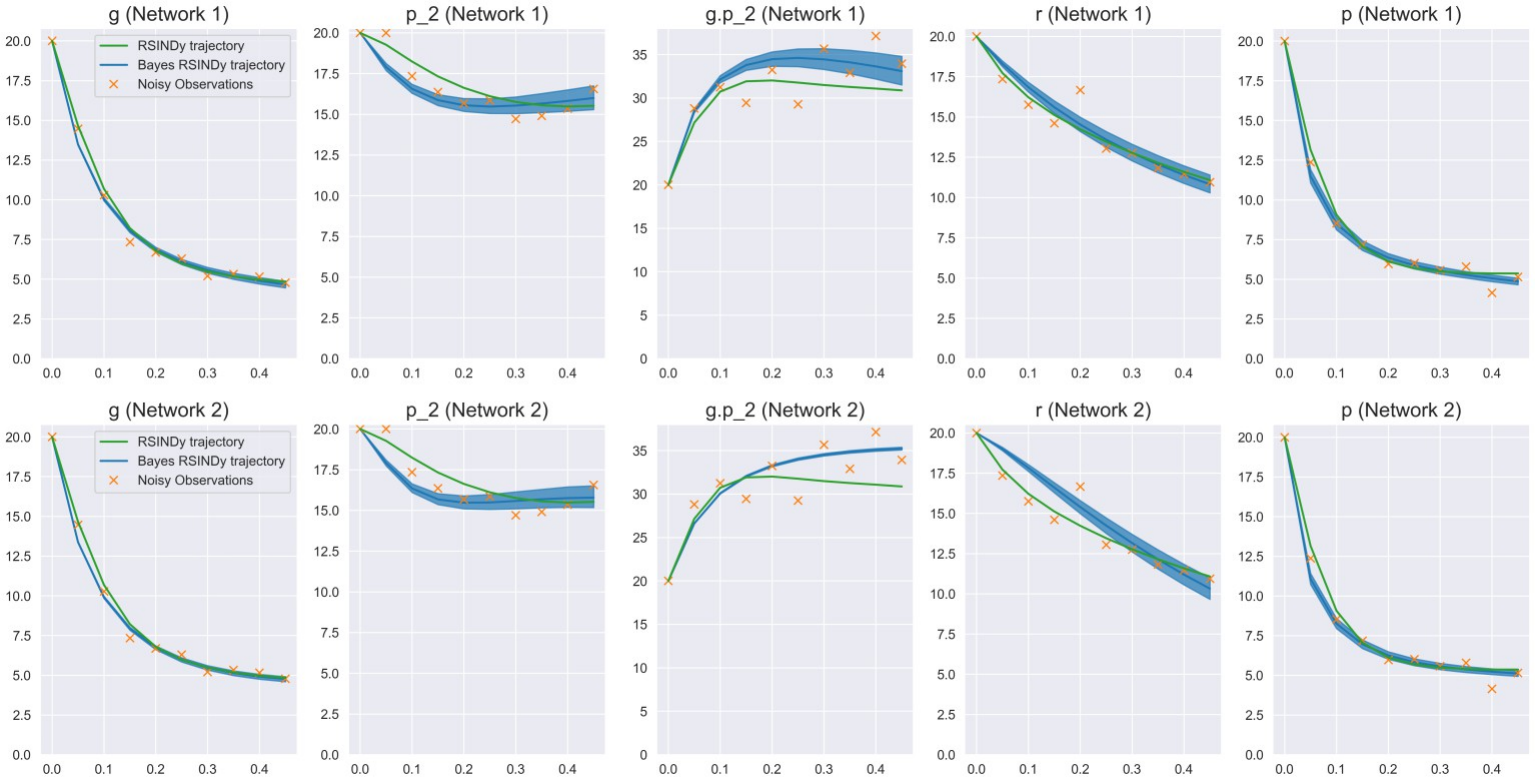
Dynamics from the identified networks. The dynamics from both recovered networks are different from the truth and each other, but still manage to produce plausible dynamics when compared to the noisy data. This points to an unidentifiability in the system, caused by noise in the data and structural identifiability issues.

**Fig 9:**
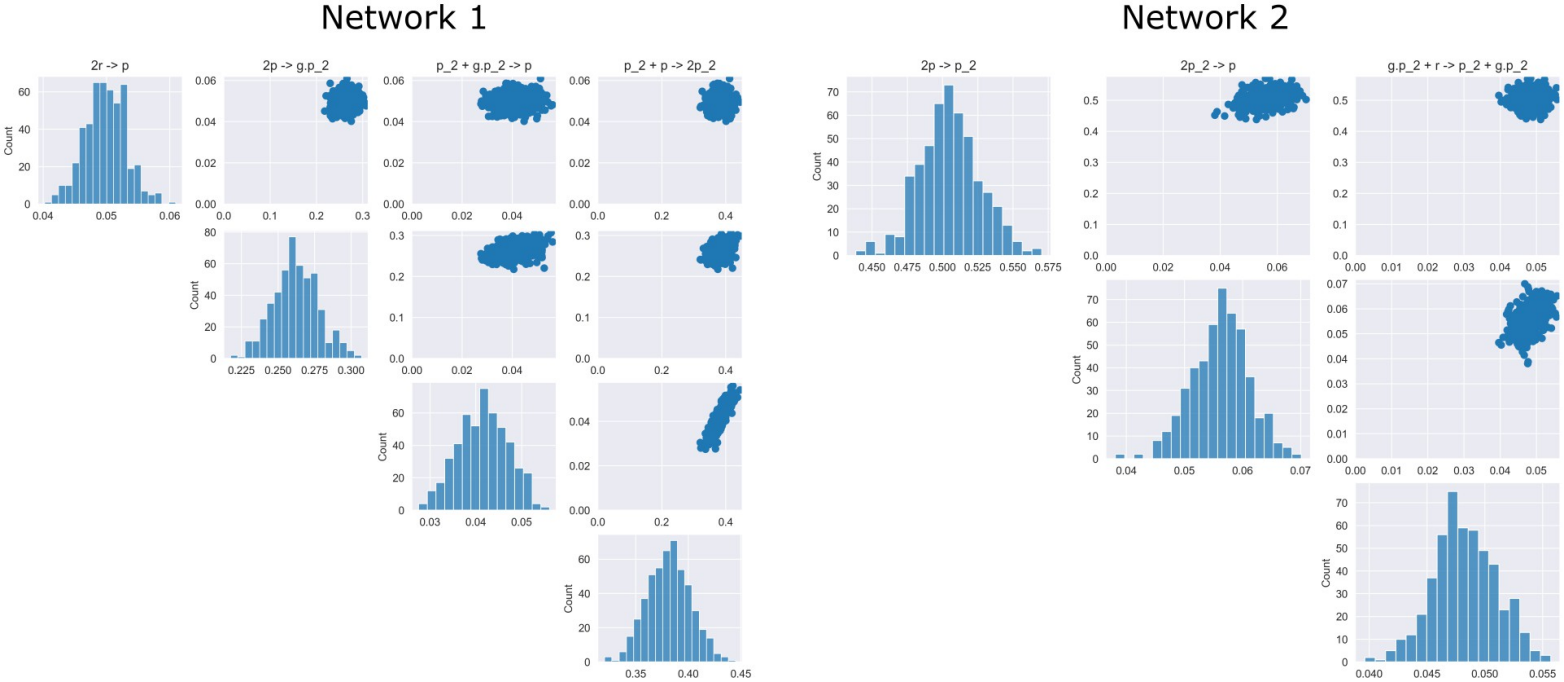
Posterior distributions over non-zero reaction rates. Pair plots of the two distinct reaction networks inferred by the model. Reaction rates within each network exhibit are relatively well determined. This indicates a distinct multi-modality or unidentifiability in the problem.

As Fig 8 demonstrates, although the reaction networks are different from the ground truth, the dynamics produced from each inferred reaction system appear plausible, especially given the noise present in data. Fig 9 shows the posterior distributions of the non-zero reactions for both networks provided by our Bayesian approach. The marginals for each reaction rate in both cases are relatively tight, indicating that the reactions are well identified within in each discovered mode.

Reactive SINDy is also capable of inferring a network, however it is considerably less sparse and with larger reaction rates than those from our method. Under a threshold of 1*e*^−2^, selected such that thresholding larger reactions changes the dynamics, the best estimated network was comprised of 24 total reactions. The full network is detailed in the S1 Appendix. As Fig 8 demonstrates, though, the replicated trajectory is still consistent with the observations. In this example, the scale of the observed concentrations and the small observation frequency provide well estimated derivatives, resulting in minimal bias for the reactive SINDy method. Under these circumstances, it may be preferable to utilize Reactive SINDy as it can be run significantly faster than our method while still providing reasonable results as shown here.

That multiple networks are obtained by different chains in this problem is largely due to the facts that our complete stoichiometric matrix constructed from the above process does not restrict many reactions. In this, an iterative procedure can be applied, where the recovered networks can be examined by the user for plausibility and implausible reactions can be excluded in future runs to converge to a different reaction system. Realistically, we expect that the complete stoichiometric matrix will often be constructed in a more careful manner so as to eliminate many of the implausible reactions before the method is used. We discuss the identifiability issue in the next section. Interestingly, the inferred networks converge largely to 2nd-order reactions to describe the system. While from a combinatorial perspective, this is not surprising considering that the ansatz library contains significantly more 2nd-order reactions than 1st-order, a possibility is to add a bias to the system for 1st-order reactions via a prior weight on certain reactions.

## Discussion

### Observational model

The latent variable formulation for the observational model provides robustness when observations are noisy or observations are not made for all of the species. In these situations, it is valid and desirable to use this model as it takes into account the true measurement process as demonstrated in the Lotka-Volterra example. However, this comes at a substantial computational cost. In some situations, when all of the species are observed and the measurements are not too noisy, simply using the Bayesian Regularized Horseshoe along with estimated derivatives as an extension to Reactive SINDy is sufficient and significantly faster to identify models. The model for this is:

$$\begin{array}{cc} \lambda_{i} & ∼{\text{Cauchy}}^{+}\left(0,1\right),\\ \tilde{\lambda_{i}} & =\sqrt{\frac{c^{2}\lambda_{i}^{2}}{c^{2}+{\left(\tau \lambda_{i}\right)}^{2}}},\\ k_{i} & ∼\text{Normal}(0,\tau \tilde{\lambda_{i}}),\;i=1,\ldots ,D.\\ \frac{d\hat{X}}{dt}\left(t_{j}\right)∼\text{Normal} & (S_{c}^{T}(\begin{array}{c} k_{1}f_{1}\left(X\left(t_{j}\right)\right)\\ k_{2}f_{2}\left(X\left(t_{j}\right)\right)\\ \cdots \\ k_{D}f_{D}\left(X\left(t_{j}\right)\right) \end{array}),1),\;j=0,\ldots ,T, \end{array}$$ (9)

where $\frac{d\hat{X}}{dt}$ is estimated numerically as previously discussed. This avoids the need to use an ODE solver and can provide Bayesian sparsity estimates similar to PTLasso [34]. We suggest that this method be used initially as it can often result in reasonable networks significantly faster.

### Identifiability

A major problem in the identification of reaction systems is the possibility of multiple structural networks which can produce nearly identical results, especially when data is limited and noisy. As multiple definitions of the unidentifiability problem can be found in the literature, in our work, we are referring to the case where different dynamic networks with different parameterizations and equations can yield indistinguishable outputs and thus cannot be identified from the data given. While the sparsity priors used in this paper aim to resolve this situation by biasing estimates toward systems with fewer reactions, this remains an issue as multiple structural pathways may still exist with a very similar number of reactions. Immediately, this issue can be somewhat relaxed in a few ways.

First, constraining the allowed reactions will naturally bias the solutions away from certain pathways. However, this requires significant domain knowledge of the species or the system under observation. The work of Tuza et al. [18] presents one possible way to restrict the reaction basis to make the problem more identifiable while also using the LASSO with estimated derivatives. Another possibility would be to first pre-process the dictionary of functions to eliminate the unidentifiable graphs aided by the concept of linearly conjugate reaction systems such as demonstrated in [38]. Further exploration in this direction is needed as their algorithm focuses on expanding a known reaction system into it’s equivalents while we do not know the reaction graph at all. This can potentially automatically eliminate the structural unidentifiabilities in the problem before inferring the system. An alternative use of the method o [38] would be to retrieve the set of unidentifiable graphs from the reaction systems inferred from our method. This would simultaneously serve as an indication of the difficulty of the inference problem as well as potentially allow the user to prune reactions based on the whole set of unidentifiable graphs. An interesting extension in this direction would be to use recent advances in Machine Learning (ML) to search the literature and generate a reasonable set of reactions given the species involved in the system [39, 40].

Alternatively, without introducing any domain knowledge, multiple MCMC chains can be used to explore all of the different networks. As each chain is trivially parallelizable, massive computational power can help explore the space more efficiently. Starting a large number of chains at different initial points will allow the chains to converge to different modalities and present it to the user as is in the case of the Prokaryotic Auto-regulation model above. A user can prune implausible reaction networks and re-run the model to converge to better solutions. The use of ML techniques such as cross-validation on a hold-out test set to automatically rank networks based on predictive accuracy [41] could be useful but limitations in the amount of data may pose a problem. Another direction that may be useful to aid in resolving unidentifiabilities is to use simulations of the retrieved networks to understand where they may deviate. If they are not structurally unidentifiable and identical everywhere, this may help to design further experiments and measurement methods to identify competing networks using new data.

As explored in Reactive SINDy, the incorporation of more data such as trajectories from multiple initial conditions can also aid in improving the identifiabiltiy of the process. Intuitively, this can be relevant in the case where certain dynamics are only present at certain concentration levels. In this case, a straightforward modification to the observational model where *L* independent trajectories are observed could be stated as follows,

$$\begin{array}{c} Z(t_{j})=\int_{0}^{t_{j}}S_{c}^{T}(\begin{array}{c} k_{1}f_{1}\left(Z\left(t\right)\right)\\ k_{2}f_{2}\left(Z\left(t\right)\right)\\ \cdots \\ k_{D}f_{D}\left(Z\left(t\right)\right) \end{array})dt\\ \hat{X_{l}}\left(t_{j}\right)∼\text{Log-Normal}(Z\left(t_{j}\right),\sigma ),\;j=0,\ldots ,T\;l=1,\ldots ,L, \end{array}$$

where $\hat{X_{l}}\left(t_{j}\right)$ refers to the observed species concentrations at time *t*_*j*_ for the *l*-th trajectory.

### Future directions & limitations

#### Scaling

As the number of species grows, the number of possible reactions grows combinatorially. This poses a significant issue computationally, as it results in a large search space for reactions and possibly further identifiability issues as demonstrated above. The scaling issue limits the practical applicability of the method to systems with a small number of active species, which we roughly estimate to be <20 based on our experiments and the amount of time that they take. While a larger set is technically possible, the computational burden may be too great to obtain results in a reasonable amount of time. One possibility is to run the method on smaller subsets of reactions to prune reactions in a sequential procedure. However, this may lead to bias issues as combining the estimates from different subsets is a non-trivial problem, especially if dealing with partial posterior distributions. Practically, biological domain knowledge can substantially help here in limiting the allowed reactions in the system or specifying known reactions as in Example 2.

Computationally, the latent variable approach with Bayesian Inference is significantly more expensive than the approach used by reactive SINDy. A large part of this is the need to compute the sensitivities of the ODE system to obtain efficient sampling. In our experiments, the auto regulatory network with 260 reactions took approximately 4 hours of time on a M1 Apple ARM processor using our approach while roughly 1.5 hours to perform a large grid search using reactive SINDy. We find that this difficulty generally scales as a function of the number of data points in addition to the number of possible reactions. A possibility on this end is to utilize Variational Inference to speed up the inference component as presented in [36]. Furthermore, there are a few different methods for computing the sensitivities of ODE systems as well as a variety of different ODE solvers [22] that may potentially offer speedups for these types of problems. For our experiments we employ the rk45 solver and a forward sensitivity solver as implemented by Stan.

#### Hyper-parameter selection of *τ*

Selection of *τ* determines the level of sparsity of these networks and, in our experience, is a pivotal hyperparameter to tune when using the horseshoe prior. Generally, we find that smaller values of *τ* will force the near-zero reaction rates to smaller values however, this typically leads to a significant decrease in computational efficiency when estimating the networks. For this reason, through our experiments and set *τ* to a small enough value such that the above pruning procedure removes a large enough set of reactions while maintaining the dynamics.

Further work exploring how to properly tune and select *τ* in a more interpretable way for reaction network inference problems is needed. A common strategy employed in other models is to place the prior *τ* ∼ Cauchy^+^(0, *τ*_0_) to allow the data to adjust *τ* [42], however this needs to be further explored in the context of the horseshoe for systems of differential equations. For linear regression models, Piironen et al. [24] propose a way to parameterize *τ*_0_ as,

$$\begin{array}{c} \tau_{0}=\frac{m_{0}\sigma }{\left(D-m_{0}\right)\sqrt{NM}}, \end{array}$$

where *m*_0_ can be derived as a guess for the effective non-zero coefficients and *σ* is the measurement noise, however our models deviate from linear regression and thus the same interpretations do not hold.

A common concern with Bayesian methods is whether the prior can be overcome with sufficient data. While in our experience, the utilized horseshoe priors are weakly informative, and indeed can be overcome with sufficient data to obtain the true network, however more rigorous study needs to be done for this. The particular case study demonstrated by Golchi et al. [43] offers good insight into the strength and importance of priors in the context of ODEs though further investigation needs to be done with respect our model and for network inference.

#### Stochastic models

Many biochemical reaction systems exhibit intrinsic stochasticity. In these situations, Eq (1) no longer sufficiently captures the dynamics of *X*(*t*) and the evolution of the system is better described using a stochastic process. While mass-action kinetics can still be applied, they now specify reaction propensities. To accommodate this, Eq (5) can be modified from the observational ODE model,

$$\begin{array}{cc} Z(t_{j}) & ∼P(Z\left(t_{j}\right)|Z\left(t_{j-1}\right),S_{c}^{T},k)\\ \hat{X}\left(t_{j}\right) & ∼P(X|Z\left(t_{j}\right)),\;j=0,\ldots ,T. \end{array}$$

where the trajectory **Z** comes from the stochastic process as specified by [44] while the regularized horseshoe and *S*_*c*_ remain as previously defined. However, the significant challenge here is that the posterior distribution becomes intractable due to the intractable likelihood term $P(Z\left(t_{j}\right)|Z\left(t_{j-1},S_{c}^{T},k\right))$, which corresponds to the solution of the chemical master equation [45]. This prevents the application of standard efficient Bayesian inference methods, which are heavily reliant on tractable likelihoods.

While there is a growing class of likelihood-free Bayesian inference methods [46] that can be applied to stochastic biochemical reaction networks, they are known to scale incredibly poorly to high dimensional parameter spaces. This makes it quite challenging to utilize with our method of network inference, which introduces a new parameter for each ansatz reaction. A possibility is to instead use stochastic approximations to the model, such as the Chemical Langevin Equation or the Linear Noise Approximation, to capture some intrinsic stochasticity, but also provide much more tractable likelihoods [44, 47, 48].

## Conclusion

In this work, we have presented a method to recover a parsimonious system of interpretable mass-action reactions directly from observations of species concentrations over time. Improving on the formulation presented by Reactive SINDy, we have modified the method via the Bayesian regularized horseshoe prior and by adapting the model as to not require derivative estimates. Our experiments show that, when identifiable, our modifications are able to recover the underlying system with uncertainty estimates from the Bayesian formulation even in sparse data scenarios. Alternatively, when unidentifiable, we present multiple sparse reaction networks which can reasonably explain the results and upon which a biologists can iterate.

## Supporting information

S1 Appendix Implementation specifics and additional experimental details and results. (PDF)

## References

[1] Abramovitch R, Tavor E, Jacob-Hirsch J, Zeira E, Amariglio N, Pappo O, et al. A pivotal role of cyclic AMP-responsive element binding protein in tumor progression. Cancer research. 2004;64(4):1338–1346. doi: 10.1158/0008-5472.CAN-03-2089 14973073

[2] Perez OD, Krutzik PO, Nolan GP. Flow cytometric analysis of kinase signaling cascades. In: Flow Cytometry Protocols. Springer; 2004. p. 67–94. 10.1385/1-59259-773-4:067 14976361

[3] Wheeler DA, Srinivasan M, Egholm M, Shen Y, Chen L, McGuire A, et al. The complete genome of an individual by massively parallel DNA sequencing. nature. 2008;452(7189):872–876. doi: 10.1038/nature06884 18421352

[4] Chen S, Mar JC. Evaluating methods of inferring gene regulatory networks highlights their lack of performance for single cell gene expression data. BMC bioinformatics. 2018;19(1):1–21. doi: 10.1186/s12859-018-2217-z 29914350 PMC6006753

[5] Margolin AA, Nemenman I, Basso K, Wiggins C, Stolovitzky G, Dalla Favera R, et al. ARACNE: an algorithm for the reconstruction of gene regulatory networks in a mammalian cellular context. In: BMC bioinformatics. vol. 7. Springer; 2006. p. 1–15. 16723010 10.1186/1471-2105-7-S1-S7 PMC1810318

[6] Leday GG, De Gunst MC, Kpogbezan GB, Van der Vaart AW, Van Wieringen WN, Van De Wiel MA. Gene network reconstruction using global-local shrinkage priors. The annals of applied statistics. 2017;11(1):41. doi: 10.1214/16-AOAS990 28408966 PMC5388190

[7] Huynh-Thu VA, Irrthum A, Wehenkel L, Geurts P. Inferring regulatory networks from expression data using tree-based methods. PloS one. 2010;5(9):1–10. doi: 10.1371/journal.pone.0012776 20927193 PMC2946910

[8] Mangan NM, Brunton SL, Proctor JL, Kutz JN. Inferring biological networks by sparse identification of nonlinear dynamics. IEEE Transactions on Molecular, Biological and Multi-Scale Communications. 2016;2(1):52–63. doi: 10.1109/TMBMC.2016.2633265

[9] Willis MJ, von Stosch M. Inference of chemical reaction networks using mixed integer linear programming. Computers & Chemical Engineering. 2016;90:31–43. doi: 10.1016/j.compchemeng.2016.04.019

[10] Morrissey ER, Juárez MA, Denby KJ, Burroughs NJ. On reverse engineering of gene interaction networks using time course data with repeated measurements. Bioinformatics. 2010;26(18):2305–2312. doi: 10.1093/bioinformatics/btq421 20639410

[11] Pan W, Yuan Y, Gonçalves J, Stan GB. Reconstruction of arbitrary biochemical reaction networks: A compressive sensing approach. In: 2012 IEEE 51st IEEE Conference on Decision and Control (CDC). IEEE; 2012. p. 2334–2339.

[12] Geurts P, et al. dynGENIE3: dynamical GENIE3 for the inference of gene networks from time series expression data. Scientific reports. 2018;8(1):1–12. doi: 10.1038/s41598-018-21715-0 29467401 PMC5821733

[13] Hoffmann M, Fröhner C, Noé F. Reactive SINDy: Discovering governing reactions from concentration data. The Journal of chemical physics. 2019;150(2):025101. doi: 10.1063/1.5066099 30646700

[14] Voit EO, Martens HA, Omholt SW. 150 years of the mass action law. PLoS Comput Biol. 2015;11(1):e1004012. doi: 10.1371/journal.pcbi.1004012 25569257 PMC4288704

[15] Hoerl AE, Kennard RW. Ridge regression: Biased estimation for nonorthogonal problems. Technometrics. 1970;12(1):55–67. doi: 10.1080/00401706.1970.10488634

[16] Tibshirani R. Regression shrinkage and selection via the lasso. Journal of the Royal Statistical Society: Series B (Methodological). 1996;58(1):267–288.

[17] Zou H, Hastie T. Regularization and variable selection via the elastic net. Journal of the royal statistical society: series B (statistical methodology). 2005;67(2):301–320. doi: 10.1111/j.1467-9868.2005.00503.x

[18] Tuza ZA, Stan GB. An automatic sparse model estimation method guided by constraints that encode system properties. In: 2019 18th European Control Conference (ECC). IEEE; 2019. p. 2171–2176.

[19] Brunton SL, Proctor JL, Kutz JN. Discovering governing equations from data by sparse identification of nonlinear dynamical systems. Proceedings of the national academy of sciences. 2016;113(15):3932–3937. doi: 10.1073/pnas.1517384113 27035946 PMC4839439

[20] Baydin AG, Pearlmutter BA, Radul AA, Siskind JM. Automatic differentiation in machine learning: a survey. Journal of machine learning research. 2018;18.

[21] Carpenter B, Hoffman MD, Brubaker M, Lee D, Li P, Betancourt M. The Stan math library: Reverse-mode automatic differentiation in C++. arXiv preprint arXiv:150907164. 2015.

[22] Chen RT, Rubanova Y, Bettencourt J, Duvenaud D. Neural ordinary differential equations. arXiv preprint arXiv:180607366. 2018.

[23] Eberhard P, Bischof C. Automatic differentiation of numerical integration algorithms. Mathematics of Computation. 1999;68(226):717–731. doi: 10.1090/S0025-5718-99-01027-3

[24] Piironen J, Vehtari A, et al. Sparsity information and regularization in the horseshoe and other shrinkage priors. Electronic Journal of Statistics. 2017;11(2):5018–5051. doi: 10.1214/17-EJS1337SI

[25] Carvalho CM, Polson NG, Scott JG. The horseshoe estimator for sparse signals. Biometrika. 2010;97(2):465–480. doi: 10.1093/biomet/asq017

[26] Ishwaran H, Rao JS, et al. Spike and slab variable selection: frequentist and Bayesian strategies. Annals of statistics. 2005;33(2):730–773. doi: 10.1214/009053604000001147

[27] Mitchell TJ, Beauchamp JJ. Bayesian variable selection in linear regression. Journal of the american statistical association. 1988;83(404):1023–1032. doi: 10.2307/2290132

[28] Neal RM, et al. MCMC using Hamiltonian dynamics. Handbook of markov chain monte carlo. 2011;2(11):2.

[29] Ranganath R, Gerrish S, Blei D. Black box variational inference. In: Artificial intelligence and statistics. PMLR; 2014. p. 814–822.

[30] Carpenter B, Gelman A, Hoffman MD, Lee D, Goodrich B, Betancourt M, et al. Stan: A probabilistic programming language. Journal of statistical software. 2017;76(1). doi: 10.18637/jss.v076.i01 PMC9788645 36568334

[31] Salvatier J, Wiecki TV, Fonnesbeck C. Probabilistic programming in Python using PyMC3. PeerJ Computer Science. 2016;2:e55. doi: 10.7717/peerj-cs.55 PMC10495961 37705656

[32] Bingham E, Chen JP, Jankowiak M, Obermeyer F, Pradhan N, Karaletsos T, et al. Pyro: Deep universal probabilistic programming. The Journal of Machine Learning Research. 2019;20(1):973–978.

[33] Breda J, Zavolan M, van Nimwegen E. Bayesian inference of the gene expression states of single cells from scRNA-seq data. bioRxiv. 2019. 10.1038/s41587-021-00875-x 33927416

[34] Gupta S, Lee RE, Faeder JR. Parallel Tempering with Lasso for model reduction in systems biology. PLoS computational biology. 2020;16(3):e1007669. doi: 10.1371/journal.pcbi.1007669 32150537 PMC7082068

[35] Hoffman MD, Gelman A. The No-U-Turn sampler: adaptively setting path lengths in Hamiltonian Monte Carlo. J Mach Learn Res. 2014;15(1):1593–1623.

[36] Ghosh S, Doshi-Velez F. Model selection in Bayesian neural networks via horseshoe priors. arXiv preprint arXiv:170510388. 2017.

[37] Wilkinson DJ. Stochastic modelling for systems biology. CRC press; 2018.

[38] Ács B, Szederkényi G, Tuza Z, Tuza ZA. Computing all possible graph structures describing linearly conjugate realizations of kinetic systems. Computer Physics Communications. 2016;204:11–20. doi: 10.1016/j.cpc.2016.02.020

[39] Ros R, Bjarnason E, Runeson P. A machine learning approach for semi-automated search and selection in literature studies. In: Proceedings of the 21st International Conference on Evaluation and Assessment in Software Engineering; 2017. p. 118–127.

[40] Radford A, Wu J, Child R, Luan D, Amodei D, Sutskever I, et al. Language models are unsupervised multitask learners. OpenAI blog. 2019;1(8):9.

[41] Vehtari A, Gelman A, Gabry J. Practical Bayesian model evaluation using leave-one-out cross-validation and WAIC. Statistics and computing. 2017;27(5):1413–1432. doi: 10.1007/s11222-016-9709-3

[42] Polson NG, Scott JG. Shrink globally, act locally: Sparse Bayesian regularization and prediction. Bayesian statistics. 2010;9(501-538):105.

[43] Golchi S. Informative priors and Bayesian computation. In: 2016 IEEE international conference on data science and advanced analytics (DSAA). IEEE; 2016. p. 782–789.

[44] Gillespie DT. The chemical Langevin equation. The Journal of Chemical Physics. 2000;113(1):297–306. doi: 10.1063/1.481811

[45] Owen J, Wilkinson DJ, Gillespie CS. Likelihood free inference for Markov processes: a comparison. Statistical applications in genetics and molecular biology. 2015;14(2):189–209. doi: 10.1515/sagmb-2014-0072 25720092

[46] Cranmer K, Brehmer J, Louppe G. The frontier of simulation-based inference. Proceedings of the National Academy of Sciences. 2020;117(48):30055–30062. doi: 10.1073/pnas.1912789117 32471948 PMC7720103

[47] Elf J, Ehrenberg M. Fast evaluation of fluctuations in biochemical networks with the linear noise approximation. Genome research. 2003;13(11):2475–2484. doi: 10.1101/gr.1196503 14597656 PMC403767

[48] Golightly A, Wilkinson DJ. Bayesian inference for stochastic kinetic models using a diffusion approximation. Biometrics. 2005;61(3):781–788. doi: 10.1111/j.1541-0420.2005.00345.x 16135029

