# Supplementary material for: Identification of dynamic mass-action biochemical reaction networks using sparse Bayesian methods
Source: PLoS Comput Biol. 2022 Jan 31;18(1):e1009830. doi: 10.1371/journal.pcbi.1009830 (PMC8830701; doi:10.1371/journal.pcbi.1009830)
Supplement: S1 Appendix — (PDF) [file pcbi.1009830.s001.pdf]

S1: Appendix

Richard Jiang Prashant Singh Andreas Hellander Linda Petzold

1 Construction of Ansatz Reactions

To construct the library of ansatz reactions used in our experiments, we use the following naive algorithm, which can be found implemented in the github repository:

1. For each species S_i, generate all reactions of type $S_i \to 0$
2. For each species S_i, generate all reactions of type, $j, k \neq i$:
   - $S_i \to S_j$
   - $S_i \to S_i + S_j$
   - $2S_i \to S_j$
   - $S_j \to 2S_i$
   - $S_i \to 2S_i$
   - $S_i \to S_j + S_k$
3. For each pair of species S_i and S_j, generate all reactions of type $k, l \neq i, j$:
   - $S_i + S_j \to 2S_i$
   - $S_i + S_j \to 2S_j$
   - $S_i + S_j \to S_k$
   - $S_i + S_j \to S_i + S_k$
   - $S_i + S_j \to S_j + S_k$
   - $S_i + S_j \to S_k + S_l$

2 Experimental Details

2.1 Lotka-Volterra Oscillator

The Lotka-Volterra Oscillator is described by

$$Y \xrightarrow{k_1} 2Y$$
$$P + Y \xrightarrow{k_2} 2P$$
$$P \xrightarrow{k_3} \phi,$$

where P represents the predator concentration in an area and Y represents the prey concentration. This is one of the simplest non-linear systems to exhibit oscillatory behavior and is often a building block for such systems. We generate data from this system by solving the corresponding ODE and then adding independent log-normal noise with standard deviation $\sigma = 0.2$.

To test our method under varying sampling frequencies, we first generate data, recording observations every $dt = 0.2$. Then, given this time series, we take every 5th observation to obtain a sampling frequency of $dt = 1$ and every 10th observation to obtain a sampling frequency of $dt = 2$.

2.1.1 Stan Model for Regularized Horseshoe of Lotka-Volterra Model

```
functions {
  vector sys(real t,
             vector y,
             vector theta) {
      vector[2] dydt;
      vector[16] v;
      matrix[2, 16] S = [
        [-2, 0, 1,-1, 0, 1,-1, 0, 0,-1, 0,-1,-2,-1, 1,-1],
        [ 0,-2, 0, 1,-1,-1, 0,-1, 1, 0,-1, 0, 1, 1,-1, 2]
      ];
      v[1] = theta[1] * y[1] * y[1];
      v[2] = theta[2] * y[2] * y[2];
      v[3] = theta[3] * y[1];
      v[4] = theta[4] * y[1]* y[2];
      v[5] = theta[5] * y[2];
      v[6] = theta[6] * y[1]* y[2];
      v[7] = theta[7] * y[1];
      v[8] = theta[8] * y[2] * y[2];
      v[9] = theta[9] * y[2];
      v[10] = theta[10] * y[1] * y[1];
      v[11] = theta[11] * y[1]* y[2];
      v[12] = theta[12] * y[1]* y[2];
      v[13] = theta[13] * y[1] * y[1];
      v[14] = theta[14] * y[1];
      v[15] = theta[15] * y[2];
      v[16] = theta[16] * y[1];

      dydt = S * v;
      return dydt;
  }
}
data {
```

```
    int N; // Number of observations
    int M; // Number of species
    int M_obs; // Observed species
    int obs_idx[M_obs]; // Indices of observed speces

    int D; // Number of possible reactions
    int D1; // Number of known rates

    vector[M] y0;
    real y[N, M_obs];
    real ts[N + 1];

    vector[D1] known_rates;

    // horseshoe parameters
    real m0;
    real slab_scale;
    real slab_df;
    real<lower = 0> tau0;


    // noise model parameters
    real<lower = 0> noise_sigma;

}

transformed data {
    real slab_scale2 = square(slab_scale);
    real half_slab_df = 0.5 * slab_df;
}

parameters {
    vector<lower = 0>[D - D1] unknown_rates_tilde;
    vector<lower = 0>[D - D1] lambda;
    real<lower = 0> c2_tilde;
}

transformed parameters {
    vector[D] rates;
    real c2;
    real tau;
    vector[D - D1] lambda_tilde;
    vector[M] y_hat[N];
    {
        tau = tau0;

        c2 = slab_scale2 * c2_tilde;

        lambda_tilde = sqrt((c2 * square(lambda)) ./ (c2 + square(tau) * square(lambda)));

        if(D1 > 0) {
          rates[:D1] = known_rates;
        }
        rates[D1 + 1:] = tau * lambda_tilde .* unknown_rates_tilde;
```

```
    }
    y_hat = ode_rk45(sys,
                     y0,
                     ts[1],
                     ts[2:],
                     rates);
}

model {
    // horseshoe priors
    unknown_rates_tilde ~ normal(0, 1);
    lambda ~ cauchy(0, 1);
    c2_tilde ~ inv_gamma(half_slab_df, half_slab_df);

    // model likelihood
    for(j in 1:M_obs) {
      y[ ,j] ~ lognormal(log(y_hat[ ,obs_idx[j]]), noise_sigma);
    }
}

generated quantities {
  real y_rep[N, M];

  for(i in 1:N) {
    for(j in 1:M) {
      y_rep[i,j] = lognormal_rng(log(y_hat[i,j]), noise_sigma);
    }
  }
}
```

2.2 Prokaryotic Auto-Regulatory Network

A simple synthetic model of auto-regulation of a protein P by a gene g in a prokaryotic cell **?** can be described using the following reaction system:

$$
\begin{aligned}
g + P_2 &\xrightarrow{k_1} gP_2 \quad \text{(Repression)} \\
gP_2 &\xrightarrow{k_2} g + P_2 \\
g &\xrightarrow{k_3} g + r \quad \text{(Transcription)} \\
r &\xrightarrow{k_4} r + P \quad \text{(Translation)} \\
2P &\xrightarrow{k_5} P_2 \quad \text{(Dimerization)} \\
P_2 &\xrightarrow{k_6} 2P \\
r &\xrightarrow{k_7} \phi \quad \text{(mRNA Degradation)} \\
P &\xrightarrow{k_8} \phi \quad \text{(Protein Degradation)},
\end{aligned}
$$

In this example, as the steady state is quickly reached, we generate synthetic data from times $t = [0, 1]$ with a sampling frequency o $dt = 0.5$. Our measurement noise model used is a lognormal error model with $\sigma = 0.07$.

3 Inferring the Prokaryotic Auto-Regulatory Network with no known reactions

Below, we present the results of our method when fitting the prokaryotic auto-regulatory network without assuming the 4 known reactions and using the same data. Interestingly, these networks are incredibly sparse while also successfully reconstructing the network.

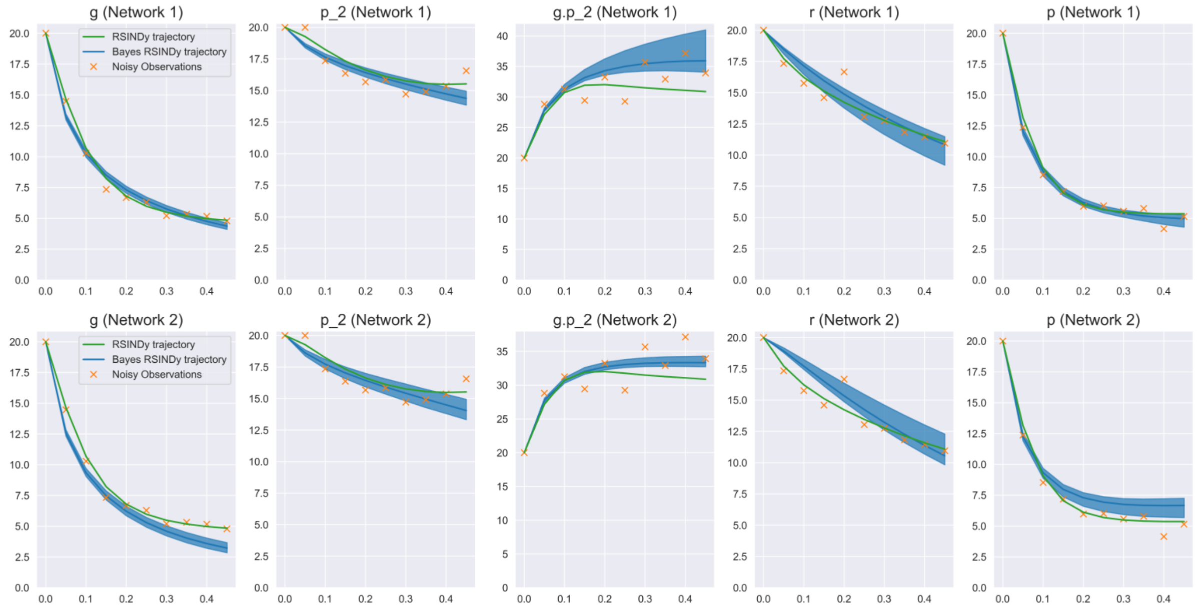

Figure 1: **Dynamics when inferring all 4 known reactions.** Similar to the case with 4 known reactions, the dynamics from both recovered networks are different from the truth and each other, but still manage to produce plausible dynamics when compared to the noisy data.

Table 1: **Selected Recovered Networks for Prokaryotic Auto-Regulation System**

True Network	Network 1	Network 2
$g + P_2 \xrightarrow{0.5} gP_2$	$2gP_2 \xrightarrow{0.005} P$	$g + P \xrightarrow{0.7} gP_2$
$gP_2 \xrightarrow{1} g + P_2$	$g + P \xrightarrow{0.66} gP_2$	$P_2 + P \xrightarrow{0.9} g + P$
$g \xrightarrow{0.15} g + r$	$P_2 + r \xrightarrow{0.8} P_2 + P$	
$r \xrightarrow{1} r + P$	$P_2 + P \xrightarrow{0.1} g$	
$2P \xrightarrow{0.5} P_2$		
$P_2 \xrightarrow{0.5} 2P$		
$r \xrightarrow{1.5} \phi$		
$P \xrightarrow{0.3} \phi$		

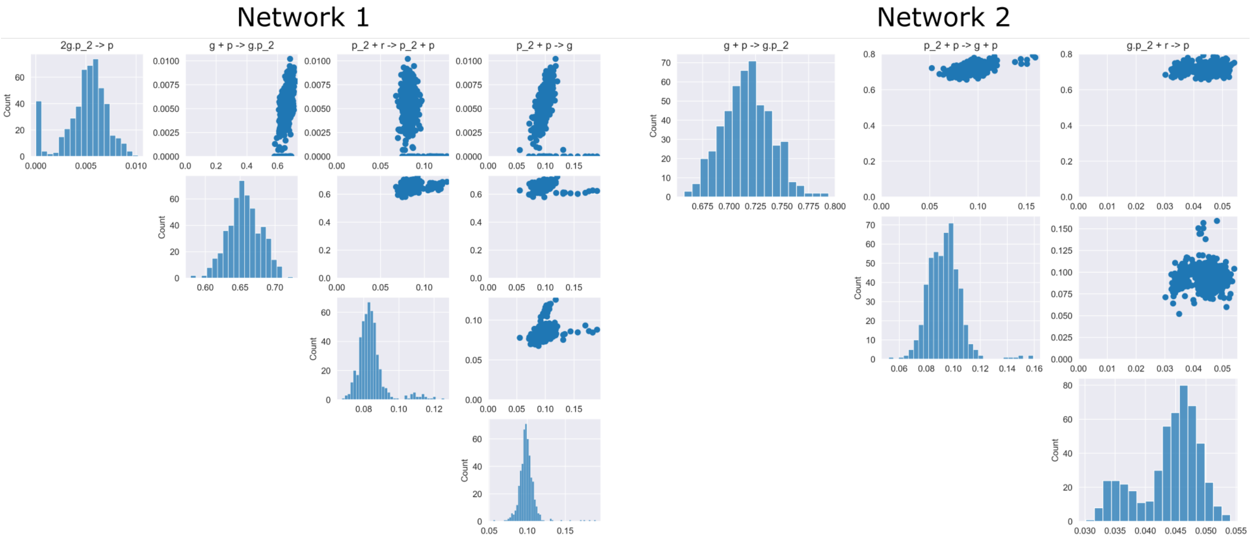

Figure 2: **Posterior Distributions over non-zero reaction rates** Pair plots of the two distinct reaction networks inferred by the model. Both largely produce similar dynamics despite the differences.

4 Network inferred by Reactive SINDy for Prokaryotic Auto-Regulation System

Table 2: **Reactive SINDy inferred Prokaryotic Auto-Regulation Network. Bolded reactions are present in the true network.**

$$\mathbf{g + P_2 \overset{0.23}{\to} gP_2}$$
$$\mathbf{r \overset{4.66}{\to} 0}$$
$$\mathbf{g \overset{7.72}{\to} 0}$$
$$P_2 \overset{6.27}{\to} 0$$
$$gP_2 \overset{8.55}{\to} 2gP_2$$
$$P \overset{1.05}{\to} P + P_2$$
$$P \overset{17.40}{\to} 2P$$
$$g + P_2 \overset{0.27}{\to} 2g$$
$$g + gP_2 \overset{0.47}{\to} 2gP_2$$
$$g + gP_2 \overset{0.05}{\to} r$$
$$g + r \overset{0.40}{\to} 2g$$
$$g + P \overset{0.03}{\to} P_2$$
$$g + P \overset{0.11}{\to} 2P$$
$$P_2 + gP_2 \overset{0.11}{\to} g$$
$$P_2 + gP_2 \overset{0.47}{\to} 2P_2$$
$$P_2 + gP_2 \overset{0.14}{\to} P$$
$$P_2 + r \overset{0.18}{\to} 2r$$
$$P_2 + r \overset{0.05}{\to} P$$
$$P_2 + P \overset{0.71}{\to} 2P_2$$
$$gP_2 + r \overset{0.03}{\to} g$$
$$gP_2 + r \overset{0.09}{\to} 2r$$
$$gP_2 + P \overset{0.18}{\to} g$$
$$gP_2 + P \overset{0.39}{\to} 2gP_2$$
$$r + P \overset{0.25}{\to} 2r$$
